# Dietary Patterns and Their Associations With the *FTO* and *FGF21* Gene Variants Among Emirati Adults

**DOI:** 10.3389/fnut.2021.668901

**Published:** 2021-05-19

**Authors:** Farah Naja, Leila Itani, Sarah Hammoudeh, Shaista Manzoor, Nada Abbas, Hadia Radwan, Maha Saber-Ayad

**Affiliations:** ^1^Clinical Nutrition and Dietetics Department, College of Health Sciences, University of Sharjah, Sharjah, United Arab Emirates; ^2^Research Institute for Medical and Health Sciences, University of Sharjah, Sharjah, United Arab Emirates; ^3^Nutrition and Food Sciences Department, American University of Beirut, Beirut, Lebanon; ^4^Department of Nutrition and Dietetics, Faculty of Health Sciences, Beirut Arab University, Beirut, Lebanon; ^5^Department of Clinical Sciences, College of Medicine, University of Sharjah, Sharjah, United Arab Emirates

**Keywords:** dietary pattern, FTO gene, FGF21 gene, western diet, traditional diet, Emirati, UAE

## Abstract

**Purpose:** To examine the dietary patterns and their associations with the FTO and FGF21 gene variants among Emirati adults.

**Methods:** Using a cross-sectional design, healthy adult male and female Emiratis (*n* = 194) were recruited from primary health care centers in Sharjah, UAE. Participants completed a 61-item semi-quantitative food frequency questionnaire. In addition, a saliva sample was obtained for the genetic analysis. Genotyping was performed for *FTOrs9939609(A*>*T), FTOrs9930506(A*>*G), FGF21 rs838133 (A* > *G)*, and *FGF21 rs838145 (A* > *G)*. Dietary patterns were derived using the principal component analysis. Logistic regression analyses were used to examine the association of dietary patterns with genetic variants.

**Results:** Three dietary patterns were identified: “Western”: consisting of fast food, sweets, and processed meat; “Traditional Emirati” rich in vegetables, traditional Emirati-mixed-dishes and whole dairy; while whole grains, low-fat dairy, and bulgur were components of the “Prudent” pattern. Subjects carrying the A allele of the *FTO rs9939609* were 2.41 times more likely to adhere to the Western pattern compared to subjects with genotype TT (OR:2.41; 95%CI:1.05–5.50). Compared with subjects with A/A, those carrying the G allele of the *FTO rs9930506* were more likely to follow a Western diet (OR: 2.19; 95%CI: 1.00–4.97). Participants carrying the risk allele (A) of the *FGF21 rs838133* were twice more likely to adhere to the Traditional pattern as compared to subjects with genotype GG (OR: 1.9, 95%CI: 1.01–3.57).

**Conclusions:** The findings of this study suggested associations among specific *FTO* and *FGF21* gene variants with dietary patterns among Emirati adults. These findings could be used to inform evidence-based targeted nutrition preventive recommendations, especially those aiming to limit intake of western type foods.

## Introduction

During the last couple of decades, obesity has become a global epidemic of major clinical and public health significance. According to the World Health Organization, obesity was ranked as the second leading cause of several non-communicable diseases (1–3). While the world is racing for the development of effective and evidence-based interventions and public health programs to halt the soaring rates of obesity, it remains paramount to have a clear understanding of the etiology of this condition.

Similar to other metabolic disorders, obesity is a complex and multifactorial disease involving genetic, environmental, and psychosocial factors (4). Up to date, a considerable amount of research has addressed these factors, however, the majority has done so in isolation. More specifically, research has been limited to addressing either genetic, environmental, or psychosocial factors while much remaining unknown about the interaction of these factors among each other. For instance, despite evidence that genetic factors play a significant role in the etiology of obesity and the large number of obesity genes identified, relatively little evidence exists about the role of these genes in altering dietary intake; the latter being largely implicated in the etiology of obesity.

Available research indicated that certain genetic variants such as the fat mass and obesity-associated (*FTO*), *MC4R, APOE*, and fibroblast growth factor −21 *(FGF21)* (5–10), which were associated with adiposity, may influence food preference patterns such as increased intake of sugar and carbohydrate consumption (11–13), total energy intake, and preferences of macronutrients (5–10). The *FTO* gene was one of the first genetic loci identified as being associated with bodyweight and strongly linked with the development of obesity (14–16). Variations in *FTO* has been associated with increased energy, fat, and protein intake (9). For instance, subjects with the *FTO rs9939609* A allele had higher carbohydrate and lower fat intake as compared to the *FTO rs9930506 GG* genotype (17). Recent research has identified a variant in the *FGF21* gene associated with preference for carbohydrate and fat intake (11). Circulating FGF21 protein levels were reported to be robustly increased with diets high in carbohydrates and low in proteins (11). In addition, elevated blood concentrations of FGF21 were positively correlated with BMI (18, 19).

The majority of available research addressing the associations of genetic factors and dietary intake has focused on single nutrients (mainly macronutrients) with very few studies addressing diet as a whole. In the real world, foods are consumed in various characteristic combinations that deliver a variety of nutrients that can have either synergistic or interactive metabolic actions. Consequently, there has been an increasing appreciation of the overall diet rather than single nutrients in examining diet-disease associations, especially for those diseases where more than one nutrient is implicated in the etiology, such is the case of obesity and other non-communicable diseases (20). In a systematic review and meta-analysis of observational studies of the association of dietary patterns with the metabolic syndrome (MetS), a prudent/healthy pattern was found to be associated with a lower prevalence of MetS, whereas a Western/unhealthy is associated with an increased risk for MetS (21). Another systematic review and meta-analysis of studies addressing dietary patterns and obesity showed that a healthy/prudent dietary pattern, consisting of vegetables, fruits, whole grains, decreases the risk of central obesity (22).

Very few studies examined the effect of individual genetic components on dietary patterns. One study reported that weight loss over 3 years among individuals following a Mediterranean diet was significantly lower in A allele carriers of *rs9939609* in the *FTO* gene compared to those homozygous for the T allele (23). Higher adherence to the Mediterranean dietary pattern using Mediterranean diet scores was associated with a decrease in obesity, regardless of *FTO* risk alleles (24).

A better understanding of the etiology of obesity and more specifically the effects of the genetic on environmental factors in decreasing/increasing its risk is especially important in countries which are witnessing alarming increasing trends of obesity and its associated non-communicable diseases. The United Arab Emirates (UAE), similar to many Gulf Cooperation Council's (GCC) countries, has witnessed an economic boom that was accompanied by rapid urbanization which lead to dietary and lifestyle changes among its population (25). These changes in dietary and lifestyle patterns contributed to an increase in diet-related chronic diseases, and obesity in all groups of the population and have consequently exerted a considerable burden on the public health sector (26).

Therefore, the aim of this study is to shed light on the effects of two genes, previously reported to be associated with obesity (*FTO* and *FGF21*) (15–17, 27, 28), on dietary patterns. More specifically the objectives of the study are to identify and characterize the dietary patterns and their correlates among a sample of UAE nationals and to examine the effect of the *FTO* and *FGF21* gene variants on adherence to the derived dietary patterns. The results of this study hold promise to increasing the efficacy of dietary approaches in the prevention of obesity and its associated chronic diseases.

## Methods

This study is a cross-sectional investigation aiming to explore the association of the (*FTO)* and *(FGF21)* variants with dietary intake among Emirati adults. Participants for this study were recruited from primary health care centers surrounding the University of Sharjah campus. To be eligible to participate in the study, subjects had to be aged 18 years and above, able to give a written consent form, and healthy (not suffering from any chronic disease, such as hypertension, diabetes mellitus or any other condition that would lead to changes in dietary intake). Participants were excluded if they had a Body Mass Index (BMI) below 16 or above 40 kg/m^2^ and if they have followed strict dietary changes in the past 2 years. The design and conduct of the project were performed according to the guidelines laid down in the Declaration of Helsinki and all procedures involving human subjects/patients were approved by the allocated ethical committee [removed for blind peer review]. All participants provided written informed consent prior to participation. A total of 194 subjects participated in this study and their data were included in the analysis. Using the OpenEpi software, this sample size allowed a power of 83% to detect prevalence difference in adherence to dietary patterns of 20% among subjects carrying and not carrying a certain allele with two sided confidence interval of 95% (29). The sample size calculation was based on the global *FTO rs9939609* minor allele frequency.

A research assistant approached visitors to the health centers in the waiting area and introduced the study with its objectives and protocol. Eligible and Interested subjects read and signed the consent form. The data collected included a saliva sample, anthropometric measurements as well as dietary intake.

For the collection of the saliva, subjects were asked if they had eaten in the last 30 min. If they did, the collection of the sample was delayed until a duration of 30 min elapsed. A volume of 2 ml of saliva samples was collected, without phlegm. The samples were preserved at −20 C0 and DNA extraction using the QIAamp extraction kit (cat# 51306) was performed within 7 days of collection. Genotyping was performed for *FTOrs9939609(A*>*T), rs9930506(A*>*G), FGF21 rs838133 (A* > *G)*, and *FGF21 rs838145 (A* > *G)* as previously described (30, 31). TaqMan1 Drug Metabolism Genotyping Assay kits were used (Applied Biosystems, USA) on the StepOne Real-Time PCR Systems platform (Thermo Fischer Scientific, USA). Allele-1 (wild) is bound to VIC, allele-2 is bound to FAM for each single nucleotide polymorphism (SNP). We used the Chi-square test to estimate Hardy–Weinberg equilibrium (32). The four SNPs were in Hardy-Weinberg equilibrium (32), Supplementary Table 1.

In a private room at the health center, anthropometry measurements including weight and height were obtained. These measurements were carried out using standardized techniques and calibrated equipment. Participants were asked to remove their shoes and any outwear. The weight was recorded to the nearest 0.1 kg. The height was measured to the nearest 0.5 cm, Using a stadiometer. Using the weight and height measurement, the BMI was calculated as weight (kg) divided by the square of height (m^2^). BMI was categorized according to the WHO classification (33) BMI <18.5 kg/m^2^ as underweight, BMI 18.5–24.9 kg/m^2^ as normal weight, BMI 25.0–29.9 kg/m^2^ as overweight, and BMI 30.0 kg/m^2^ or greater as obese (18).

The dietary intake of participants was examined using a semi-quantitative sixty-one-item Food Frequency Questionnaire (FFQ). Participants were asked to recall their intake over the past year. The FFQ was designed by a panel of experts (including a nutritionist, an epidemiologist and a public health nutritionist) and aimed to examine food consumption of Emirati adults. The questionnaire consisted of three components: the food list, the portion size, and the frequency options. The food list included foods and beverages commonly consumed by Emirati adults. In order to assist the participants in estimating the portion size, he/she was given three option: 1- use a standard portion, expressed in household measures, 2- select the reference portions as listed in the two-dimensional food portion visual (Millen and Morgan, Nutrition Consulting Enterprises, Framingham, Massachusetts, United States), or use the supplementary visual aids about portion sizes of common items in the traditional Gulf and Middle Eastern cuisine meals [Abu Dhabi Food Control Authority. A Photographic Atlas of Food Portions for the Emirate of Abu Dhabi. User'sGuide. Abu Dhabi: 2014. Abu Dhabi Food Control Authority]. For the frequency section, participants were asked to indicate the frequency of consumption either per day, per week, per month, per year or never. The reported frequency of each food item and beverage was later converted to a daily portion intake. In order to calculate the energy, macronutrient, and micronutrient consumption by participants, the dietary intake data derived from the FFQ was analyzed using NUTRITIONIST PROTM diet analysis software (Axxya Systems LLC., USA, version 5.1.0, 2014, First Fata Bank, Nutritionist Pro, San Bruno, CA). Given the lack of local Emirati food composition database, the USDA database was used in the estimation of energy, macro, and micro nutrients of dietary intake.

### Derivation of Dietary Patterns

Principle component analysis was used to derive the dietary patterns. For this purpose, the 61 food items listed in the FFQ were grouped into 18 food groups based on culinary usage and common nutrient composition (Supplementary Table 2). Certain food items had a unique composition [eggs, olives, Burghol (parboiled wheat)] and hence were classified individually. The consumption from each food group was calculated as the sum of total gram intake per day from all items included in this group. Exploratory Principle Component Analysis (PCA) was conducted using total gram intake for each food group. The suitability of data for running PCA was examined using the Bartlett test of sphericity and the Keiser-Mayer-Olkin test which were considered significant with a *p* < 0.05 and a score > 0.6, respectively. The number of factors retained was based on the first inflection point of the scree plot and interpretability of the factors. Orthogonal transformation of the retained factors was used to maximize variances of the squared loadings and improve the interpretability of the patterns. Derived patterns were labeled based on food groups with factor loading > 0.4. factor scores were calculated using a regression approach where each individual received a score on each pattern. The calculated pattern score reflected the degree of adherence of an individual to a specific dietary pattern, with higher scores indicating higher adherence.

Data analysis was conducted using IBM SPSS statistics software version 25 for Windows. Descriptive characteristics were presented for continuous variables as mean ± standard deviation (SD) and for categorical variables as frequency and percentage. The correlations of the scores of each identified pattern with energy, macro- and micro nutrients were carried out using Pearson correlations and statistical significance of the difference between dependent correlations was tested for using Steiger's Z formula (34). The correlation analysis was based on energy adjusted intake for micronutrients using residual method (35). In order to examine the association of the derived dietary patterns with the various genetic variants, simple and multiple logistic regression analyses were conducted. The outcomes variables in these logistic regression models were adherence to the patterns, defined as having a pattern score greater than the median value. The main independent variables were the genetic variants of both the *FTO* and *FGF21* genes. In the multiple regression, adjustment was carried for age, sex, BMI and energy intake. Simple and multiple logistic regression analyses were also conducted to test for the associations of adherence to the dietary patterns with age, sex and BMI. A p-value lower than 0.05 was considered statistically significant.

## Results

Table 1 summarizes the descriptive characteristics of the study population as well as their genotype distribution for four selected SNPs: *FTO rs9939609* (risk allele A), *FTO rs9930506* (risk allele G), *FGF21 rs838133* (risk allele A), and *FGF21 rs838145* (risk allele G) (Table 1).

**Table 1:** Descriptive characteristics of the study population (*n* = 194).

**Characteristics**		**N(%) or mean ± SD**
Age (years)		30.38 ± 9.75
Sex	Females	109 (56.19)
	Males	85 (43.81)
BMI (kg/m^2^) (Continuous)		26.6 ± 5.82
BMI ^¶^ (Categorical)	Underweight	11 (5.67)
	Normal weight	67 (34.54)
	Overweight	64 (32.99)
	Obese	52 (26.8)
Energy (Kcal)		3517.6 ± 1269.69
**Genes and risk allele**		
*FTO rs9939609*	AA	31 (15.98)
	A/T	81 (41.75)
	TT	73 (37.63)
	Missing	*9 (4.64)*
*FGF rs838133*	AA	22 (11.34)
	A/G	71 (36.6)
	GG	100 (51.55)
	Missing	*1 (0.52)*
*FTO rs9930506*	G/G	40 (20.62)
	HA/G	78 (40.21)
	AA	67 (34.54)
	Missing	*9 (4.64)*
*FGF rs838145*	GG	14 (7.22)
	A/G	77 (39.69)
	AA	102 (52.58)
	Missing	*1 (0.52)*

*BMI, Body Mass Index. Data are expressed as mean ± SD for continuous variables and frequency and percentage for categorical variables*.

¶ *BMI was stratified according to the WHO criteria*.

**Figure 1:**
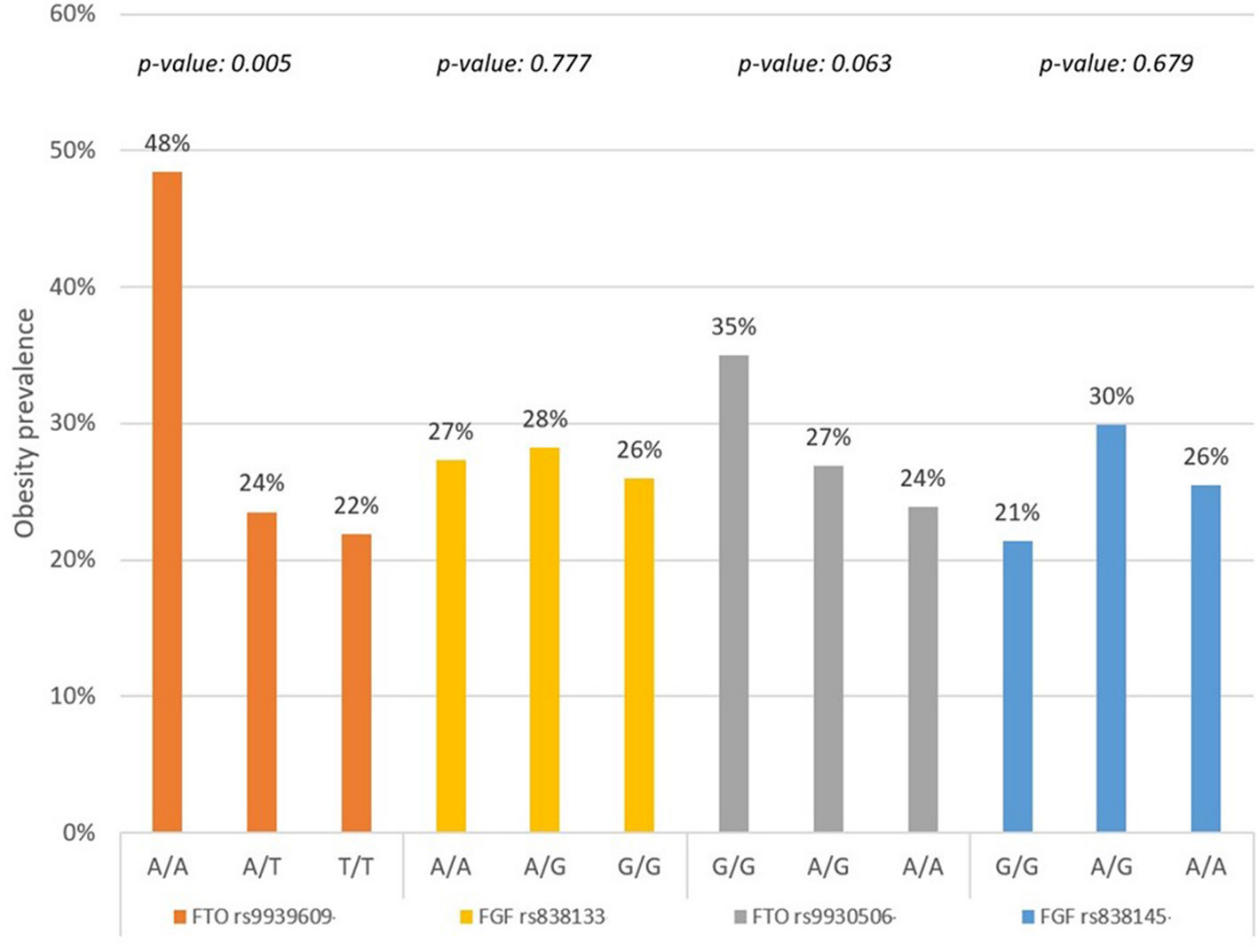
Prevalence of obesity by gene and risk allele in the study population (*n* = 194).

Figure 1 describes the prevalence of obesity (BMI ≥ 30.0 kg/m^2^) by allele in the study population. For both genes, the *FTO* and *FGF21*, the prevalence of obesity was highest for the homozygous risk alleles, except for the *FGF21 rs838145*, where the highest obesity prevalence was observed among the heterozygous A/G (Figure 1).

**Figure 2:**
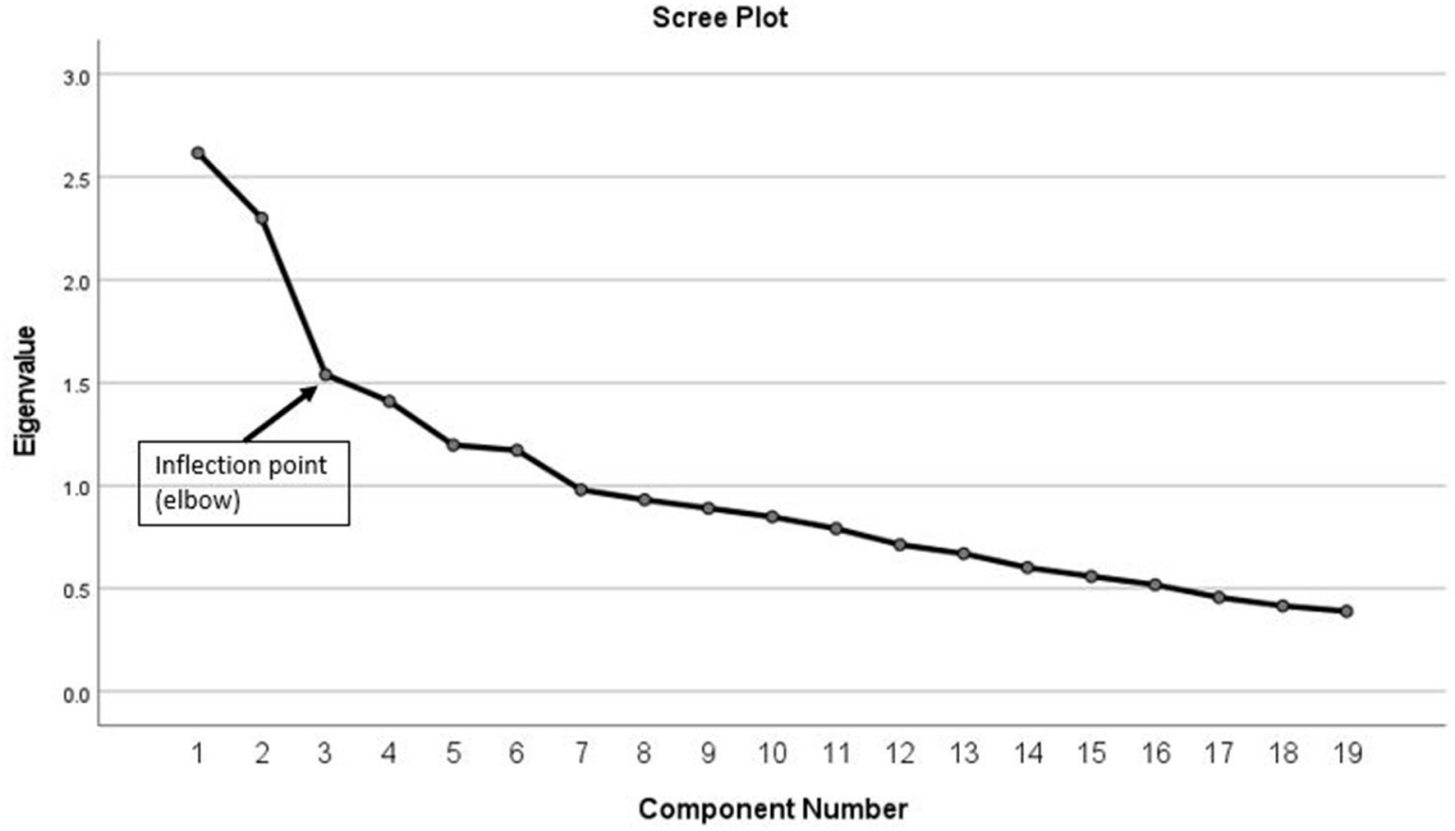
Scree plot, as derived from the principal component analysis (PCA) of the dietary intake among study population (*n* = 194).

Figure 2 shows the scree plot as derived from the PCA of the dietary intake among the study population with an inflection at the third component suggesting three patterns of dietary intake.

The factor loadings of the various food groups and their corresponding patterns derived from the PCA in the study population are presented in Table 2. After examination of these loadings, the three patterns were names as Western, Traditional Emirati and Prudent. The Western pattern consisted of the following food groups: fast food, sweets, processed meat, fats and oils, sugar-sweetened beverages and refined cereals. The food groups included in the “Traditional Emirati” pattern were vegetables, traditional Emirati mixed dishes, fruits, whole milk and dairy products, nuts and seeds, eggs, and olives. Whole grains, low fat milk and dairy products, meat, and bulgur were components of the “Prudent” dietary pattern (Table 2).

**Table 2:** Factor loadings of the various food groups and their corresponding patterns, as derived from Principal Component Analysis (PCA) in the study population (*n* = 194).

	**Dietary patterns**		
	**Western**	**Traditional**	**Prudent**
Fast foods	0.692		−0.116
Sweets	0.678		
Processed meat	0.592		
Fats and oils	0.568		
Sugar sweetened beverages	0.549		−0.121
Refined grains	0.378	0.143	−0.192
Water	−0.124		
Vegetables	−0.275	0.709	
Traditional Emirati mixed dishes		0.697	
Fruits	0.197	0.474	0.134
Whole milk and dairy products	0.160	0.431	−0.365
Nuts and seeds	0.185	0.333	
Eggs		0.316	
Olives		0.281	
Whole grains	−0.205		0.736
Low fat milk and dairy products	−0.239		0.707
Meat (red meat, fish, and poultry)	0.171	0.281	0.659
Bulgur		0.299	0.380
Variance explained (%)	12.5	11.6	9.8

*Extraction Method: Principal Component Analysis*.

*Factor loadings <0.1 were removed for simplicity*.

The association of energy, macro- and micro- nutrients with the derived patterns in the study population is summarized in Table 3. The highest correlations with energy and fat intakes were observed for the Western pattern. On the other hand, the Traditional Emirati pattern had the highest correlation with carbohydrates while the scores of the Prudent diet had the highest correlation with proteins. The Western dietary pattern had significant negative correlations with most of the micronutrients studied (vitamin A, vitamin D, vitamin B9, calcium, iron and sodium). The highest association with sodium was observed for the Traditional pattern (Table 3).

**Table 3:** Association of energy, energy adjusted macro- and micro nutrients with the derived patterns in the study population (*n* = 194)^€^.

**Energy and nutrients**	**Western**	**Traditional**	**Prudent**
Energy (Kcal)	0.716^**^a	0.494^**^b	0.125
Carbohydrates (g)	−0.004	0.198^**^a	−0.260^**^b
Proteins (g)	−0.287^**^a	0.061	0.138^**^b
Fats (g)	0.381^**^a	0.002	−0.199
Vitamin A (ug RAE)	−0.293^**^a	0.524^**^b	0.141^*^c
Vitamin C (mg)	−0.115	0.467^**^	0.070
Vitamin D (ug)	−0.156^*^a	0.242^**^b	0.268^**^b
Vitamin B9 (ug)	−0.333^**^a	0.588^**^b	0.270^**^c
Calcium (mg)	−0.392^**^a	0.193^**^b	0.118
Iron (mg)	−0.398^**^a	0.140	0.240^**^b
Sodium (mg)	−0.321^**^a	0.615^**^b	−0.001
Zinc (mg)	0.142^*^a	−0.024	0.318^**^b

€ *Energy adjustment was carried out using residual method (35)*.

* *Correlations are significant at p < 0.05*.

** *Correlation significantly different at P < 0.01*.

*^a, b, c^Values with different superscripts are significantly different p < 0.05 (Using the Steiger's Z formula to test the statistical significance of the difference between dependent correlations)*.

The associations between age, sex, and BMI with the identified dietary patterns were examined and results are summarized in Table 4. Older participants were less likely to follow a western diet and males were more likely to follow the traditional dietary pattern. BMI was significantly associated with the traditional dietary pattern score only: overweight participants were more likely to follow a traditional diet as compared to normal weight participants (Table 4).

**Table 4:** Association of age, sex, and BMI with the identified dietary patterns in the study population (*n* = 194).

		**Western Score**			**Traditional Score**			**Prudent Score**		
		**(Above median vs. Below median)**			**(Above median vs. Below median)**			**(Above median vs. Below median)**		
		**OR**	**95%CI**	***p-*value**	**OR**	**95%CI**	***p-*value**	**OR**	**95%CI**	***p-*value**
Age		0.94	(0.91–0.97)	** <0.001**	1.01	(0.98–1.04)	0.531	1.02	(0.99–1.05)	0.214
Sex	Females	–			–			–		
	Males	1.34	(0.76–2.37)	0.312	1.88	(1.06–3.35)	**0.031**	1.34	(0.76–2.37)	0.312
BMI^¶^	Underweight	2.16	(0.53–8.87)	0.284	2.44	(0.65–9.13)	0.186	0.76	(0.21–2.74)	0.677
	Normal	–			–			–		
	Overweight	0.59	(0.3–1.18)	0.137	2.17	(1.08–4.37)	**0.029**	0.91	(0.46–1.81)	0.798
	Obese	0.75	(0.36–1.55)	0.439	1.1	(0.53–2.3)	0.790	0.85	(0.41–1.75)	0.653

*BMI, Body Mass Index; OR, Odds Ratio; CI, Confidence Interval*.

¶ *BMI was stratified according to the WHO criteria. p-values in bold are statistically significant (<0.05)*.

Table 5 summarizes multiple logistic regression analyses for the association of dietary patterns with various genotypes adjusted for age, sex, BMI and energy. For *FTO rs9939609*, compared to homozygous TT, carriers of the A allele (homozygous or heterozygous form) had a higher adherence to the Western pattern. Similarly, for the *FTO rs9930506*, participants with the G allele (GG or AG) were more likely to adhere to this pattern, compared to homozygous AA genotype. As for the Traditional Emirati pattern, carriers of the A allele of *FGF rs838133* were more adherent in comparison to participants who have the homozygous GG genotype (Table 5).

**Table 5:** Multiple logistic regression for the associations between the various genotypes and their alleles with the identified dietary patterns in the study population (*n* = 194)^*^.

		Adherence to dietary patterns^**^								
**Gene and risk allele**		**Western pattern**^**a**^			**Traditional pattern**^**b**^			**Prudent pattern**^**c**^		
		**OR**	**95%CI**	***p-*value**	**OR**	**95%CI**	***p-*value**	**OR**	**95%CI**	***p-*value**
*FTO rs9939609*	AA & A/T	2.41	(1.05; 5.50)	**0.037**	0.88	(0.46; 1.7)	0.707	1.23	(0.67; 2.24)	0.510
	TT (reference)	1			1			1		
*FGF rs838133*	AA & A/G	0.65	(0.31; 1.39)	0.268	1.9	(1.01; 3.57)	**0.04**	1.59	(0.89; 2.85)	0.118
	GG (reference)	1			1			1		
*FTO rs9930506*	GG & A/G	2.19	(1.00; 4.97)	**0.05**	1.07	(0.55; 2.1)	0.835	0.96	(0.52; 1.79)	0.909
	AA (reference)	1			1			1		
*FGF rs838145*	GG & A/G	0.95	(0.45; 2)	0.889	1.39	(0.74; 2.6)	0.309	1.37	(0.77; 2.45)	0.282
	AA (reference)	1			1			1		

*BMI, Body Mass Index; OR, Odds Ratio; CI, Confidence Interval*.

*p-values in bold are statistically significant (<0.05)*.

* *Adjustment was carried out for Adjusted for age, sex, BMI, and energy*.

** *Adherence is defined as having a score greater than the median of a particular pattern*.

a *Western score median: −0.2276477*.

b *Traditional score median: −0.0063727*.

c *Prudent score median: −0.234393*.

## Discussion

The current study was conducted to explore the link between *FTO* and *FGF21* gene variants and dietary patterns in the Emirati population. The findings of this study revealed three dietary patterns in the sample population, namely the Western, Traditional Emirati, and Prudent patterns. Significant associations were observed between the *FTO* gene variants and the Western pattern as well as between the *FGF21* gene variants and the Traditional Emirati dietary pattern. The Traditional Emirati pattern had the highest correlation with carbohydrates and sodium while the scores of the Prudent diet had the highest correlation with proteins. On the other hand, the highest correlations with energy and fat intakes were observed for the Western dietary pattern as compared to the Traditional Emirati and the Prudent patterns.

The three patterns identified in this study are commonly reported in other studies of dietary patterns in the literature. For instance, a national study in Lebanon, aiming to examine dietary patterns and their association with obesity among adults also identified similar three patterns (36). Another multi-center case control study in Spain derived three comparable patterns among a sample of 2,034 post-menopausal women; namely Western, Prudent and Traditional Mediterranean (37). A more recent study in the Uruguay examining dietary patterns in association with prostate cancer also identified similar three patterns, in addition to a substituier and a drinker patterns (38). Although the specific foods contributing to each pattern may vary in their level of contribution among various studies, a few common traits characterized these patterns. For instance, the Western pattern generally refers to diets high in fat, red and processed meat, high-fat dairy products, and refined cereals. The prudent diet, also named healthy pattern in many studies, is often marked by the consumption of fruit and vegetables, whole grains, and fish (39).

Compared to the western and prudent diets, less consistent definitions exist for the Traditional dietary patterns across the literature. As the name depicts, this pattern is rather descriptive of the ethnic and country-specific food consumption pattern. For instance, while a traditional diet may hold traits of a Mediterranean pattern in some countries of the Mediterranean basin such as Spain and Lebanon, it may not be the case in other countries. In this study, the traditional pattern refers to the pattern endogenous to the Emirati population consisting of traditional Emirati mixed dishes, vegetables and fruits, and whole milk and dairy. The mixed dishes consist primarily of rice and meat (red meat or poultry). A similar traditional pattern was also reported by Al-Assa et al. (40). In a 3-day record of diet in 170 Saudi adult males, Al-Assa et al. (40) reported the composition of the traditional diet as consisting of mutton, white rice, wheat bread, and dates; chicken, camel meat, tomatoes, cucumber, potatoes, broad beans, and watermelon; cultured buttermilk, sweet black tea and Arabic coffee. In the Arabian Peninsula, traditional diets have consisted primarily of dates, wheat, barley, rice, and meat; as well as yogurt products, such as leben.

In the current study, we studied two SNPs in the *FTO* gene and two SNPs in *FGF21* gene. The allele frequencies in the studied Emirati cohort was closest to that reported for the South Asian and European populations (Supplementary Table 3). The UAE has broadly a high heterogeneous population (41), with a high Asian component due to immigration from geographically close countries and social relationships (42). There may be also a European component through Portuguese influence during the seventeenth century due to colonialization, trading and subsequent social relationships (43). In general, genetic features of Emirati population are shared with other Arabian Peninsula populations (44).

The findings of this study revealed significant effects of specific genes on dietary intakes, as participants carrying the risk allele (A) of the *FGF21 rs838133*were twice more likely to adhere to the Traditional pattern as compared to subjects with genotype GG. The Traditional Emirati dietary pattern had the highest correlation with carbohydrates, compared to the Western and Prudent patterns. Previous research suggested the *FGF21* genetic variants are associated with food preference, affecting primarily with dietary carbohydrate intake (27, 45). In fact, a significant association was reported by Søberg et al. between *FGF21 rs838133* and increased consumption of sweet (31). In further support to our findings, Hill et al. (46) indicated in his review of FGF21 and its influence on the physiological regulation of macronutrient preference that circulating FGF21 levels are robustly increased by diets that are high in carbohydrate. In our study, the Traditional diet, associated with *FGF21 rs838133 A* allele, was also associated with high sodium intake. Such an association confirmed the findings of an earlier investigation by our group indicating a high preference for salty food among Emiratis (47). In line with the evidence for a higher sodium consumption for subjects carrying the *FGF21 rs838133 A* allele, Frayling et al. reported that this gene variant is associated with high blood pressure (48). Noteworthy, a genome-wide association study showed the *FGF21 rs838133* as the strongest locus for urinary sodium (49). Therefore, targeted interventions should be directed to limit the simple carbohydrate and sodium content of Traditional Emirati dishes and be replaced with complex high fiber carbohydrate alternatives. Special attention ought to be dedicated to Emiratis with *FGF21 rs838133 A* allele when planning precision and individualized nutrition intervention.

In the current study, *FTO* gene variants were significantly associated with the Western pattern, whereby carriers of the risk alleles for either *rs9939609* or *rs9930506* were 2.41, and 2.19 times, respectively, more likely to have a Western score above the median. The Western pattern in this study was found to have the highest association with energy and fat intake. Variants of the *FTO* were suggested as a risk factor for obesity in genome-wide association studies (50) Previous studies addressing the effect of the *FTO* on dietary intake also showed that variations in *FTO* were associated with increased energy and fat intake (51, 52). In fact, a combined analysis of 16,094 participants from 14 studies showed that the *FTO* variant was associated with increased total energy intake (53).

Allelic variants in *FTO* are assumed to raise obesity risk through impaired central nervous system satiety processing, thereby increasing caloric intake (54). Based on our analysis, none of the studied gene variants were significantly associated with the Prudent dietary pattern score.

An interesting finding in this study is the coexistence of the three dietary patterns among participants: Western, Traditional Emirati and Prudent. The concomitant presence of these patterns further highlights the nutrition transition that the UAE is experiencing. The term “nutrition transition” has been predominantly used to characterize the shifts observed in dietary intake, whereby traditional diets are slowly eroding to be replaced more Western pattern diets high in sugars and fat and animal-source food (55). These shifts in dietary patterns have been found to largely coincide with an epidemiological transition of diseases with high rates of obesity, diabetes, and overall, non-communicable diseases (55). In this study, older participants were more likely to be adherent to the Traditional Emirati diet as compared to the younger participants; further supporting the notion of nutrition transition in the country. Previous studies have also found an inverse association between age and adherence to Western type of diets (56–60). It is suggested that globalization, taking place in many countries in the world as well as in the UAE, plays a major role in accentuating this transition. In fact, globalization affects the nature of the food supply chain, thereby altering the quantity, type, cost, and desirability of foods available for consumption (61). Although previous studies have addressed the associations of genetic variants with dietary intake (17, 62) in the MENA region, the current study is the first to describe the link of gene variants and dietary patterns. Using the pattern approach, as opposed to single nutrients or foods approach, to examine dietary intake in this study offered a holistic assessment of intake and produced recommendations that are easily translated into public health messages. In addition, dietary intake was assessed using an interviewer-based culture-specific food frequency questionnaire, hence providing an estimation of long term dietary intake. That said, a few limitations ought to be considered in the interpretation of the results of this study. First, the analytical process of the PCA for the derivation of dietary patterns necessitates a few subjective decisions among which is the examination of the scree plot in determining the number of factors to extract (63). In this study, while six factors (patterns) had an eigenvalue > 1, only three were above the breaking point in the scree plot and were retained in the analysis. Previous studies have also used the point of inflection in the scree plot, in addition to an eigenvalue greater than one, in deciding on the number of factors to be retained (64–66). Similar to the results of this study, such an approach yielded a fewer number of factors to be retained as compared to relying solely on the criteria related to the eigenvalue. Second, the limited sample size available hindered the examination of the effects of each of the homozygous and heterozygous risk allele separately, hence future larger studies are needed to confirm our findings. Third, The FFQ used in the present study was not previously validated among Emirati adults; however, this FFQ is adapted from another questionnaire which was used for the assessment of dietary patterns and their relationship with obesity and the metabolic syndrome among adults from another Arab country (Lebanon), the results of which are published elsewhere (57, 67). To overcome the possible differences in dietary intake between Lebanese and Emirati dietary intake, an expert panel, consisting of a dietician and a nutrition epidemiologist examined the original food list of the FFQ and adapted it to the Emirati culture, including the traditional and native foods and dishes.

## Conclusions

Our study identified three distinct dietary patterns in a cohort of adult Emiratis. The role of *FTO* and *FGF21* gene polymorphisms was explored in relation to these patterns, whereby the *FTO* and *FGF21* gene variants were associated with higher adherence to the Western and Traditional diets, respectively. As such, based on the results of this study, nutrition preventive recommendations and programs aiming to limit adherence to the Western dietary pattern could target Emirati adults carrying the A risk alleles for *FTO rs9939609* and the G risk alleles for *FTO rs9930506*. Though preliminary, these findings support the concept of precision nutrition, whereby dietary recommendations are individually tailored to prevent adherence to certain dietary patterns associated with chronic diseases on the basis of genomic background. Future research addressing the impact of genes on dietary habits and metabolites are needed to prevent and manage diseases with complex etiologies such as obesity and non-communicable diseases.

## Data Availability Statement

The datasets presented in this study can be found in online repositories. The names of the repository/repositories and accession number(s) can be found in the article/Supplementary Material. The raw data supporting the conclusions of this article will be made available by the authors, without undue reservation.

## Ethics Statement

The studies involving human participants were reviewed and approved by the approval of the University of Sharjah Research and Ethics Committee (REC-19-05-27-02) was obtained before we started subject recruitment. The patients/participants provided their written informed consent to participate in this study.

## Author Contributions

FN, HR, and MS-A conceived the idea of the study and developed the overall plan. SM and SH carried out the practical and laboratory work. HR supervised the administration and performed the analysis of the FFQ. MS-A supervised the genotyping. FN, LI, and NA carried out the PCA and other statistical analyses. FN, HR, and MS-A wrote the manuscript. All authors contributed to the article and approved the submitted version.

## Conflict of Interest

The authors declare that the research was conducted in the absence of any commercial or financial relationships that could be construed as a potential conflict of interest.
