# Supplementary material for: Dietary Patterns and Their Associations With the FTO and FGF21 Gene Variants Among Emirati Adults
Source: Front Nutr. 2021 May 19;8:668901. doi: 10.3389/fnut.2021.668901 (PMC8171665; doi:10.3389/fnut.2021.668901)
Supplement: Supplementary file 1 [file Data_Sheet_1.docx]

Supplementary Material

**Table S1:** Hardy-Weinberg equilibrium testing and context sequences of gene variants

All p-values are > 0.05, consistent with Hardy-Weinberg equilibrium

| Gene variant | X^2^ | p-value | Context sequence |
| --- | --- | --- | --- |
| FTO *rs9939609* | 0.59 | 0.44 | GGTTCCTTGCGACTGCTGTGAATTT[A/T]GTGATGCACTTGGATAGTCTCTGTT |
| FGF21 *rs838133* | 1.45 | 0.23 | ACGAGACCGGGTTCGAGCACTCAGG[A/G]CTGTGGGTTTCTGTGCTGGCTGGTC |
| FTO *rs9930506* | 1.92 | 0.17 | AGGGACACAAAAAGGGACATACTAC[A/G]TGAATTACTAATATCTAAGAAAATA |
| FGF21 *rs838145* | 0.005 | 0.94 | ATTGCCAGCCGAGGATAGGGAAAAC[A/G]GTATTTACTAGCCTCGGGGAACCTC |

**Table S2:** items included in the food groups used in the PCA.

| **Food groups** | **Items** |
| --- | --- |
| Fast foods | Pies Manaeesh, Falafel Sandwich, Chawarma Sandwich, Hamburger, Pizza |
| Sweets | Cakes, Cookies, Doughnut, Muffin, Croissant, Ice-Cream, Chocolate, Honey, Jam, Sugar, Molasses, Arabic Sweets |
| Processed meat | Luncheon Meat, Sausages Hotdogs, Makanek |
| Fats and oils | Butter, Mayonnaise, Vegetable Oil |
| Sugar sweetened beverages | Nescafe, Tea, Sugar Sweetened Beverages, Coffee, Cacoa |
| Refined grains | Pasta Cooked, Bread White, Breakfast Cereals |
| Water | Water |
| Vegetables | All vegetables (Fresh, canned and frozen) |
| Traditional Emirati mixed dishes | Rice and Rice Based dishes common to the UAE. |
| Fruits | All fruits, Dried Fruits, Fruit Juice Fresh |
| Whole milk and dairy products | Whole Dairy Product: Milk, Yogurt, Labneh, Cheese |
| Nuts and seeds | Nuts and Seeds Roasted Salted |
| Eggs | Eggs |
| Olives | Olive |
| Whole grains | Bread Whole Wheat, Breakfast Cereals Bran |
| Low fat milk and dairy products | Low Fat Dairy (Milk Half Skimmed, Cheese Low Fat, Yogurt Low Fat) |
| Meat (red meat, fish and poultry) | Meat Fish and Poultry Products |
| Bulgur | Cooked bulgur |

| Gene variant | Allele | | Frequency | | | | | | |
| --- | --- | --- | --- | --- | --- | --- | --- | --- | --- |
|  |  |  | EMI | ALL | AFR | AMR | EAS | EUR | SAS |
| *FTO rs9939609* | | A | 0.39 | 0.34 | 0.51 | 0.26 | 0.17 | 0.41 | 0.29 |
|  |  | T | 0.61 | 0.66 | 0.49 | 0.74 | 0.83 | 0.59 | 0.71 |
| *FGF21 rs838133* | | A | 0.30 | 0.23 | 0.26 | 0.25 | 0.006 | 0.43 | 0.19 |
|  |  | G | 0.70 | 0.77 | 0.74 | 0.75 | 0.994 | 0.58 | 0.81 |
| *FTO rs9930506* | | G | 0.43 | 0.29 | 0.17 | 0.26 | 0.22 | 0.44 | 0.4 |
|  |  | A | 0.57 | 0.71 | 0.83 | 0.74 | 0.78 | 0.56 | 0.6 |
| *FGF21 rs838145* | | G | 0.27 | 0.16 | 0.02 | 0.23 | 0.004 | 0.42 | 0.2 |
|  |  | A | 0.73 | 0.84 | 0.98 | 0.77 | 0.996 | 0.58 | 0.8 |
|  | |  |  |  |  |  |  |  |  |
| Emirati Population in the current study showed allele frequencies of *FTO (rs9939609 and rs9930506) and FGF21 (rs838133 and rs838145)* close to South Asians and Europeans.  EMI: Emirati population in the current study, ALL: average global frequency, AFR: African, AMR: American, EAS: East Asian, EUR: European, SAS: South Asian. Data retrieved from the “GWAS Catalog” (Buniello A, MacArthur JAL, Cerezo M, Harris LW, Hayhurst J, Malangone C, McMahon A, Morales J, Mountjoy E, Sollis E, Suveges D, Vrousgou O, Whetzel PL, Amode R, Guillen JA, Riat HS, Trevanion SJ, Hall P, Junkins H, Flicek P, Burdett T, Hindorff LA, Cunningham F and Parkinson H. The NHGRI-EBI GWAS Catalog of published genome-wide association studies, targeted arrays and summary statistics 2019. Nucleic Acids Research, 2019, Vol. 47 (Database issue): D1005-D1012) | | | | | | | | | |

**Table S3:** Allele Frequencies of the studied *FTO (rs9939609 and rs9930506) and FGF21 (rs838133 and rs838145)* in different population
